# Comprehensive computational analysis of deleterious nsSNPs in *PTEN* gene for structural and functional insights

**DOI:** 10.22099/mbrc.2025.52148.2092

**Published:** 2025

**Authors:** Divyanshi Sharma, Harasees Singh, Aryan Arya, Himanshi Choudhary, Pragya Guleria, Sandeep Saini, Chander Jyoti Thakur

**Affiliations:** 1 Department of Bioinformatics, Goswami Ganesh Dutta Sanatan Dharma College, Sector 32C, 160030, Chandigarh, India; 2 Department of Biophysics, Panjab University, Sector 25, 160014, Chandigarh, India

**Keywords:** nsSNP, MD Simulation, Cancer, PTEN, Protein Modelling, Structural Alignment

## Abstract

Single nucleotide polymorphisms (SNPs) are pivotal in understanding the genetic basis of complex disorders. Among them, nonsynonymous SNPs (nsSNPs) that alter amino acid sequences can significantly impact protein structure and function. This study focuses on analyzing deleterious nsSNPs in the tumor suppressor gene *PTEN* (Phosphatase and TENsin Homolog), which plays a central role in regulating the PI3K/Akt signaling pathway and tumorigenesis. Out of 43,855 SNPs in *PTEN*, 17 deleterious nsSNPs were identified using six computational tools. Protein stability analysis revealed that 15 variants reduce stability, potentially leading to functional impairment. Structural evaluations using HOPE and ConSurf classified mutations into buried structural residues disrupting protein integrity and exposed functional residues affecting molecular interactions. STRING database analysis highlighted PTEN as a central node in an intricate protein network, with deleterious mutations impairing critical interactions with partners such as PIK3CA, AKT1, and TP53. Secondary structure analysis revealed distinct structural deviations, particularly for G129E, which exhibited the most pronounced destabilization. Molecular dynamics simulations confirmed stability variations across mutants, with G129E exhibiting greater instability. This comprehensive analysis enhances understanding of *PTEN* nsSNP impacts, offering insights for therapeutic interventions and future experimental validation.

## INTRODUCTION

Cancer is one of major the global concern. A study conducted by GLOBCAN (Global Cancer Observatory) estimates that approximately 9.7 million people died from cancer in 2022, and by the end of 2025, that figure is predicted to increase to 10.5 million. By 2050, an estimated 35 million more instances of cancer are anticipated [1]. The understanding cancer at the genetic level is important, as the diversity and nature of genetic variation in specific genes significantly influence the initiation, progression, and therapeutic outcome of the diseases [2-4]. In this regard, one of the gene is *PTEN* which is crucial tumor suppressor that has been vastly investigated among the many genes linked to the genesis of cancer [5-7]. *PTEN* gene, located on chromosome 10 and spanning 121 kilobases of DNA across 9 exons, encodes a protein that serves as a critical regulator in the phosphatidylinositol-3-kinase (PI3K)/ Akt signaling pathway. This pathway is integral to cellular processes such as growth, survival, and angiogenesis. In response to receptor tyrosine kinase (RTK) activation, PI3K catalyzes the conversion of phosphatidylinositol (4,5)-bisphosphate (PIP2) into phosphatidylinositol (3,4,5)-trisphosphate (PIP3), a key signaling lipid that activates the serine/threonine kinase Akt. Hyper-activation of Akt, often due to elevated PIP3 levels, has been implicated in the pathogenesis of various cancers. PTEN functions as a vital tumor suppressor by dephosphorylating PIP3, thereby attenuating Akt signaling and maintaining cellular homeostasis [8-13].

Genetic alterations in the *PTEN* gene are frequently observed in patients with brain tumors. Mutations in *PTEN* can lead to protein dysfunction, disrupted cellular signaling, and tumorigenesis. PTEN is associated with a spectrum of developmental disorders collectively referred to as PTEN Hamartoma Tumor Syndrome (PHTS), which includes conditions such as Cowden syndrome and Lhermitte-Duclos disease. Individuals with these syndromes are at a significantly elevated risk for various malignancies, including breast (lifetime risk: 85%), thyroid (lifetime risk: 38%), kidney (renal cell carcinoma; lifetime risk: 34%), and endometrial (lifetime risk: 28%) tumors, which may be either benign or malignant [14-19].

Many of the genetic variations observed in the *PTEN* gene are attributed to single nucleotide polymorphisms (SNPs), the mutations occurring at individual base pairs. SNPs account for approximately 90% of genetic variation in the human genome. When these nucleotide substitutions result in an amino acid change in the encoded protein, they are classified as non-synonymous SNPs (nsSNPs). nsSNPs are particularly significant because they can influence protein stability and function, potentially leading to altered cellular behaviour, adverse drug responses, and an increased risk of hereditary disorders [20, 21].

Our study aimed to analyse the all nsSNP related to *PTEN* gene by employing in silico approach which can provide valuable insights into the functional consequences of these mutations. Similar in silico studies were conducted previously which used a limited number of tools for analysing different types of cancer in correlation to *PTEN* [22]. But a comprehensive study is yet to be conducted on total nsSNPs of *PTEN* gene so our study tries to refine the understanding of the structural and functional effects of mutations in the *PTEN* gene by using a wider array of tools and methods to analyse an extensive set of 1434 nsSNPs.

## MATERIALS AND METHODS

Figure S1 shows the workflow of methodology.

### Data Acquisition of nsSNP:

We have retrieved the dataset of nsSNP from dbSNP (https://www.ncbi.nlm.nih.gov/snp/). A total of 1434 nsSNPs of the human *PTEN* gene were obtained through the dbSNP database. The FASTA sequence of the *PTEN* was acquired from the UniProt database (https://www.uniprot.org/). 

### Detection of Deleterious nsSNPs:

The prediction of deleterious nsSNPs were conducted using six computational tools: SIFT (Sorting Intolerant from Tolerant), Missense3D, SNP&GO, PolyPhen2 (Polymorphism Phenotyping v2), PANTHER (Protein Analysis Through Evolution-ary Relationships), and FATHMM (Functional Analysis through Hidden Markov Models). SIFT (https://sift.bii.a-star.edu.sg/) predicted the amino acid substitution based on sequence homology and physical properties of amino acids. Scores below 0.05 were classified as deleterious, whereas scores above 0.05 were considered tolerable [23]. Missense3D (http://missense3d.bc.ic. ac.uk/) evaluated the structural impact of missense variations by comparing experimental and predicted protein structures, distinguishing between damaging and neutral variation [24]. SNP & GO (https://snps-and-go.biocomp.unibo.it/snps-and-go/) assessed the pathogenicity of single point mutations using support vector machine (SVM), classifying mutations as pathogenic when scores exceeding 0.5 [25]. PolyPhen2 (http://genetics.bwh.harvard.edu/pph2/) evaluated the functional impact of amino acid substitutions, categories them as as benign, possibly damaging, or probably damaging [26]. PANTHER (https://www.pantherdb.org) predicted whether observed amino acid substitution at a specific position will be functionally neutral or deleterious [27]. FATHMM (https://fathmm.biocompute.org.uk/) assigned the functional implications of nsSNPs with scores above 0.5 signifing potential damage, whereas a score below 0.5 considered benign or neutral [28].

### Prediction of Protein Stability:

 The impact of nsSNP on protein stability was assesses using I-Mutant 2.0, INPS-MD (Impact of Non-synonymous mutations on Protein Stability - Multi Dimension), and MUpro. I-MUTANT (https://folding.biofold.org/i-mutant/i-mutant2.0. html) employed SVM model. To predict alterations in protein stability resulting from single point mutation, providing the stability changes in terms of ΔΔG values [29]. INPS-MD (https://inpsmd.biocomp.unibo.it) classified mutation as stabilizing (ΔΔG>0) or destabilizing (ΔΔG<0) based on sequence or 3D protein structure input [30]. MUpro (http://mupro. proteomics.ics.uci.edu/) employed SVM and neural networks to determine whether single site amino acid mutations increased or decreased protein stability based on a positive or negative scores [31].

### Analysis of Protein Properties:

 Protein properties were analyzed using HOPE (Have Your Protein Explained) (https://www3.cmbi.umcn.nl/hope/) which assessed the impact of point mutations on the structural conformation and function of proteins. HOPE evaluated the inconsistencies between the amino acids in the wild type and the variants to predict their effects [32].

### Conservational Analysis:

Evolutionary conservation of amino acid positions was assessed using ConSurf database (https://consurfdb.tau.ac.il/) which assigned a conservation scores ranging from 1 to 9. Scores of 1 indicated rapidly evolving (variable), while the scores of 5 and 9 represented mildly evolving and conserved positions, respectively [33].

### Protein-Protein Interaction Network Analysis:

STRING (Search Tool for the Retrieval of Interacting Genes/Proteins) (https://string-db.org/) was used to analyze protein-protein interaction networks. It conducted the analysis through seven channels: neighborhood, gene fusion, co-occurrence, co-expression, experiments, databases, and text-mining [34].

### Conserved Domain Analysis:

The conserved domains were identified using the Conserved Domain Database (CDD) (https://www.ncbi.nlm.nih.gov/cdd/), which employes Reverse Position Specific BLAST (RPS-BLAST) to identify protein sequences with conserved domain footprints and functional locations [35]. 

### Secondary Structure Prediction:

The secondary structure of PTEN and its variants were predicted using PSIPred (Position Specific Iterated-BLAST-based secondary structure PREDiction) (http://bioinf.cs.ucl.ac.uk/psipred/). This tool applies PSI-BLAST to generate position-specific scoring matrices (PSSMs) and two-stage neural network to refine protein secondary structure [36]. 

### 3D protein modelling of Wild type and Mutants:

 The tertiary structure of PTEN and its variants was modeled using I-TASSER (Iterative Threading ASsembly Refinement) (https://zhanggroup.org/I-TASSER/). It begins with an amino acid sequence and uses iterative structure assembly simulations and numerous threading alignments to create three-dimensional (3D) atomic models [37, 38]. The modeled structures were refined by a web based server, GalaxyWEB (https://galaxy.seoklab.org/cgi-bin/submit.cgi?type=REFINE) based on CASP9 (9th Critical Assessment of techniques for protein Structure Prediction [39]. The built models were then validated by PROCHECK Ramachandran plot analysis (https://saves.mbi.ucla.edu/), which estimated structural quality by analyzing bond angles, torsion angles [40]. Protein structural similarity between the modelled wild-type and mutant protein was assessed by TM-align (Template Modelling Align) (https://zhanggroup.org/TM-align/), which computes the Root-Mean-Square Deviation (RMSD) and TM-score, ranging from 0 to 1. Proteins with a TM-score above 0.5 are generally considered to belong to the same fold, whereas those with a TM-score below 0.5 are less likely to share the same fold. A TM-score of less than 0.17 is an indication of structurally unrelated proteins [41].

### Molecular Dynamics (MD) Simulation:

 MD simulation was conducted using WebGRO (http://simlab.uams.edu/) for a 50 ns period, including energy minimization, equilibration, molecular dynamics, and trajectory analysis analysis. The resulting simulated model was analyzed using a number of parameters, including RMSD (Root Mean Square Deviation), RMSF (Root Mean Square Fluctuation), Rg (Radius of Gyration), SASA (Solvent-Accessible Surface Area), and the average number of hydrogen bonds per frame over time [42].

## RESULTS

The SNPs of the *PTEN* gene were obtained from the dbSNP database. The collected 43,855 SNPs included 1,434 non-synonymous SNPs, 593 synonymous SNPs, and 37,758 intronic SNPs, while the remainder falls into miscellaneous groups (Supplementary Fig. S2).

For our analysis, 1,434 nsSNPs were subjected to comprehensive functional characterization using six advanced in silico tools, namely, SIFT, Missense3D, SNP&GO, FATHMM, Polyphen-2, and PANTHER. The six analytical tools showed variations in the prediction of deleterious nsSNPs. We used a consensus approach to increase the reliability of the prediction by taking into consideration all the tools reporting consistently predicted deleterious. The nsSNPs that were consistently classified as deleterious across multiple tools were defined as high-risk nsSNPs, as they are more likely to have a significant impact on protein structure and function based on computation prediction. The following substitutions, identified as deleterious through this consensus approach, were selected seventeen nsSNPs, M35R, H61D, L70P, H93R, D107N, L112P, H123R, C124R, G129E, R130G, R130Q, G132V, I135T, S170R, R173C, R173H, and D252G for further analysis (Table 1).

**Table 1:** Selection of high risk nsSNPs.

**SNP ID’s**	**Variants**	**SIFT**	**Missense** **3D**	**SNP&GO**	**FATHMM**	**POLYPHEN2**	**PANTHER**
rs121909225	M35R	Deleterious	Damaging	Disease	Pathogenic	PD	PD
rs121909236	H61D	Deleterious	Neutral	Disease	Pathogenic	PD	PD
rs121909226	L70P	Deleterious	Damaging	Disease	Pathogenic	PD	PD
rs121909238	H93R	Deleterious	Neutral	Disease	Pathogenic	PD	PD
rs57374291	D107N	Deleterious	Neutral	Disease	Pathogenic	PD	PD
rs121909230	L112P	Deleterious	Damaging	Disease	Pathogenic	PD	PD
rs121909222	H123R	Deleterious	Neutral	Disease	Pathogenic	PD	PD
rs121909223	C124R	Deleterious	Damaging	Disease	Pathogenic	PD	PD
rs121909218	G129E	Deleterious	Neutral	Disease	Pathogenic	PD	PD
rs121909224	R130G	Deleterious	Damaging	Disease	Pathogenic	PD	PD
rs121909229	R130Q	Deleterious	Damaging	Disease	Pathogenic	PD	PD
rs121909241	G132V	Deleterious	Damaging	Disease	Pathogenic	PD	PD
rs370795352	I135T	Deleterious	Neutral	Disease	Pathogenic	PD	PD
rs121909221	S170R	Deleterious	Damaging	Disease	Pathogenic	PD	PD
rs121913293	R173C	Deleterious	Neutral	Disease	Pathogenic	PD	PD
rs121913294	R173H	Deleterious	Neutral	Disease	Pathogenic	PD	PD
rs121909239	D252G	Deleterious	Damaging	Disease	Pathogenic	PD	PD

Note: PD=probability damaging

The impact of individual amino acid changes on protein stability was assessed using MUpro, INPS-MD, and I-Mutant. These tools calculate the free energy change value (DDG) and the scale indicates the direction in which the DDG will either rise or decrease throughout the evaluation. The DDG value indicates protein stability, with a positive value indicating an enhancement in protein stability, whereas a negative score indicates an expected decrease in protein stability. Therefore, the substitutions M35R, H61D, L70P, H93R, D107N, L112P, C124R, G129E, R130G, R130Q, G132V, I135T, R173C, R173H and D252G were thus interpreted as decreasing the protein stability by all the three tools (Table 2) and considered for further analysis. Whereas, mutant H123R and S170R indicate increase in energy and these variants were not pursued for further analysis in this study, as our primary focus was on destabilizing mutations that may be implicated in disease phenotypes.

**Table 2:** Impact of nsSNPs on the stability of protein

**Variants**	**MU-PRO**		**INPS-MD**			**I-Mutant**	**Consensus**
	**DDG**	**Prediction**	**DDG** **(INPS sequence)**	**DDG** **(INPS-3D)**	**Prediction**	**Prediction**	
M35R	-1.596	Decrease	-0.54704	-1.2152	Decrease	Decrease	Decrease
H61D	-1.08	Decrease	-0.91993	-1.35091	Decrease	Decrease	Decrease
L70P	-2.364	Decrease	-3.12543	-3.27085	Decrease	Decrease	Decrease
H93R	-0.367	Decrease	-0.58974	-0.51096	Decrease	Decrease	Decrease
D107N	-0.483	Decrease	-0.47412	-0.75478	Decrease	Decrease	Decrease
L112P	-1.817	Decrease	-3.1771	-3.24848	Decrease	Decrease	Decrease
C124R	-1.212	Decrease	-1.31188	-1.95472	Decrease	Decrease	Decrease
G129E	-0.54	Decrease	-1.05818	-1.07509	Decrease	Decrease	Decrease
R130Q	-0.719	Decrease	-1.16329	-1.00918	Decrease	Decrease	Decrease
R130G	-1.332	Decrease	-0.92267	-1.53274	Decrease	Decrease	Decrease
G132V	-0.364	Decrease	-1.54026	-0.74085	Decrease	Decrease	Decrease
I135T	-1.697	Decrease	-2.89611	-2.98536	Decrease	Decrease	Decrease
R173C	-1.327	Decrease	-0.40444	-0.78835	Decrease	Decrease	Decrease
R173H	-1.854	Decrease	-1.09851	-1.35065	Decrease	Decrease	Decrease
D252G	-1.771	Decrease	-0.42007	-0.87011	Decrease	Decrease	Decrease

Project HOPE combines the characteristics of standard and altered amino acids, such as size, charge, and hydrophobicity, to forecast how mutations affect the structure and function of proteins. HOPE analysis showed that mutant residues H61D, L70P, L112P, R130G, I135T, R173C, R173H, D252G are of smaller size compared to their respective wild-type residues, while mutant residues M35R, H93R, D107N, C124R, G129E, G132V show increased size. In addition, we propose that these mutations impact the structure of proteins, specifically in areas near a highly conserved region (Supplementary Table S1).

**Figure 1:**
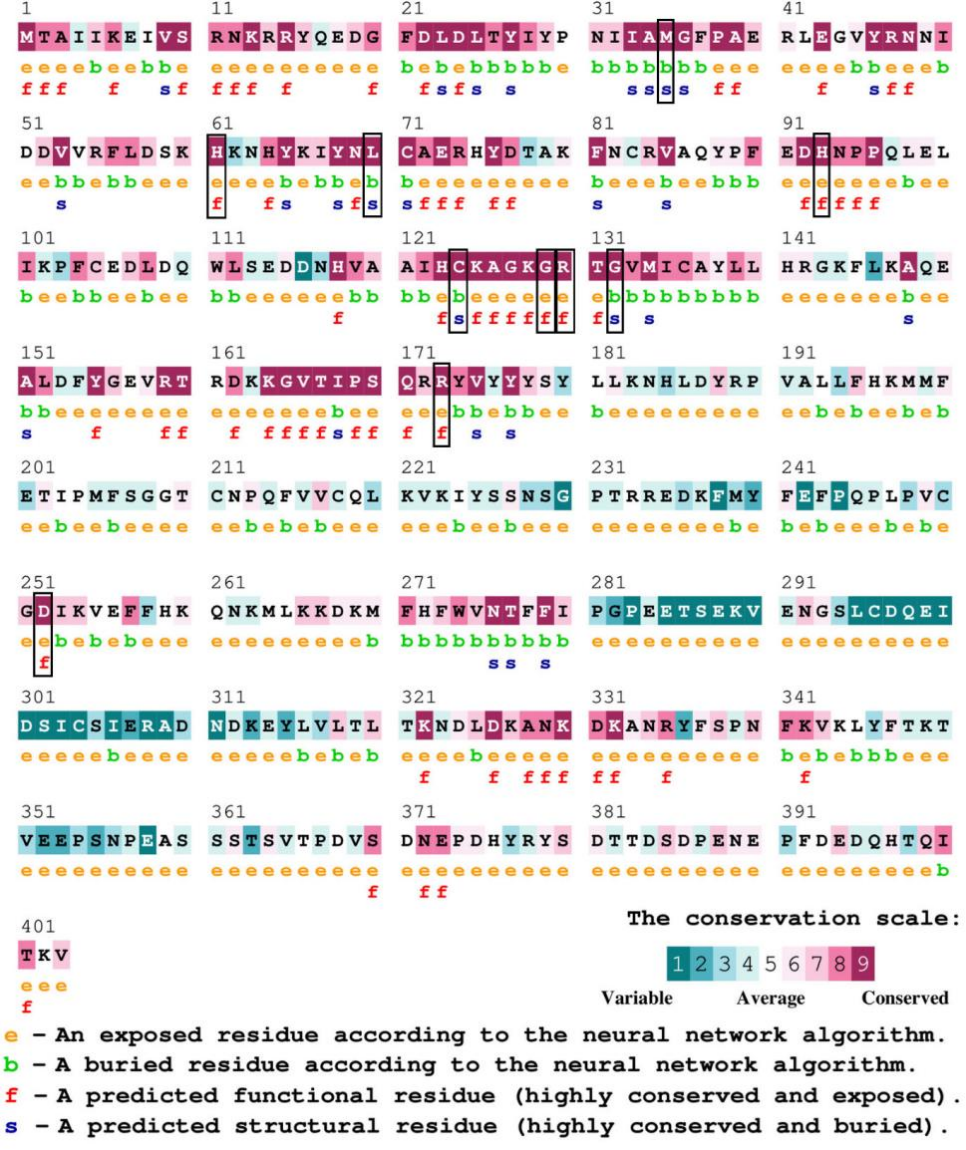
Conservation analysis of PTEN by using ConSurf. Mutations with a conservation of score nine are highlighted in the black box border.

The evolutionary conservation of the amino acid residues in the PTEN protein has been examined using the ConSurf server. Among the 15 most detrimental mutations, twelve (M35R, H61D, L70P, H93R, C124R, G129E, R130G, R130Q, G132V, R173C, R173H, and D252G) were assessed to be well conserved, achieving a conservation value of 9, while the rest three mutations obtained a conservation score ranging from 7 to 8. The mutants with conservation scores of 9 were chosen for additional investigation (Fig. 1).

The STRING analysis found that PTEN interacts with key cellular signaling and cancer development proteins. Major functional partners include TP53, a key tumor suppressor gene involved in cell cycle arrest and apoptosis, and MAGI2, which appears to function at synaptic junctions. AKT1 is a critical PI3K/Akt pathway component regulating survival and growth confidence. PIK3CA and PIK3R1, components of phosphatidylinositol 3-kinase and an upstream regulator of AKT activation; PTK2 is associated with cell adhesion and death; SPOP and PREX2 are involved in ubiquitin signaling; and DLG1, MAGI2, and MAST2 are scaffold proteins that control cell signaling and structure (Supplementary Fig. S3). 

**Figure 2:**
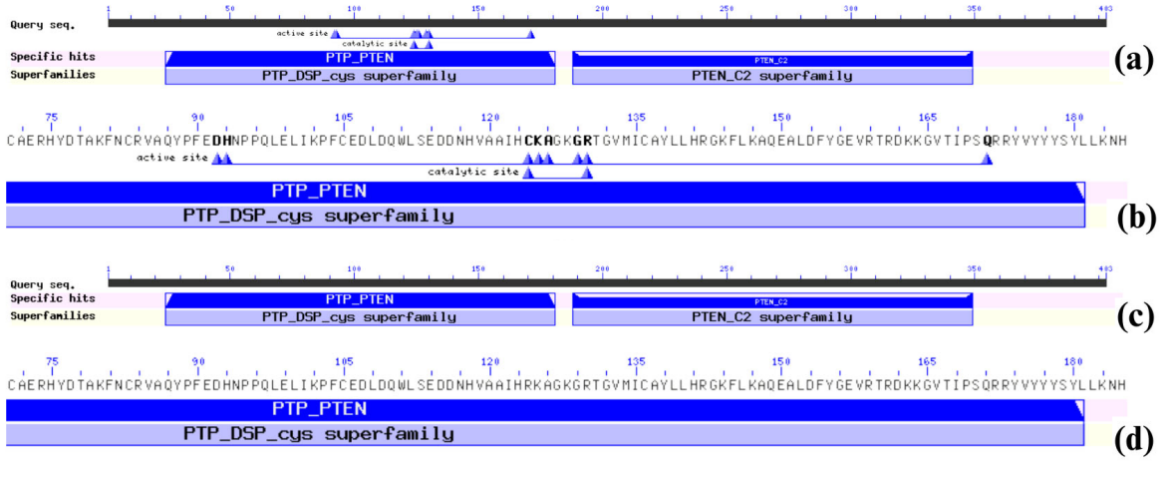
Conserved Domain Architecture of wild-type PTEN and its mutants: (a) Conserved domain architecture of wild-type PTEN, showing the PTP_PTEN and PTEN_C2 domains along with active and catalytic sites. (b) A zoomed-in view of the PTP_PTEN domain from (a), highlighting specific residues involved in the active and catalytic sites. (c) Conserved domain analysis of PTEN variants C124R, R130G, and R130Q, showing alterations in domain integrity. (d) A zoomed-in view of the PTP_PTEN domain from (c), illustrating the loss of active and catalytic site residues due to mutations.

The Conserved Domain Database (CDD) identified the PTP_PTEN and PTEN_C2 domains as the functional domains of the wild-type PTEN tumor suppressor protein (Fig. 2a). A zoomed-in view of the PTP_PTEN domain (Fig. 2b) highlights key residues involved in the active and catalytic sites. The active site consists of Asp192, His193, Cys124, Lys125, Ala126, Gly129, Arg130, and Gln171, which are crucial for PTEN's enzymatic function. The catalytic site includes Cys124 and Gly129, which play essential roles in substrate binding and catalysis. However, PTEN mutants C124R, R130G, and R130Q exhibited a loss of these active and catalytic sites (Fig. 2c). A zoomed-in view (Fig. 2d) further illustrates the absence of these critical residues, which may impair PTEN’s normal functionality. In contrast, the remaining mutants retained the same domain architecture and active/catalytic site pattern as the wild-type PTEN.

Using PSIPRED, we analyzed the secondary structures of both wild-type PTEN and its variants, focusing on the composition of helices, strands, and coils. The wild-type PTEN serves as a baseline, comprising nine helices, sixteen strands, and twenty-five coils. Variants such as C124R and R130G exhibit noticeable change deviations. C124R has increased in strands (eighteen) and coils (twenty-seven), while R130G has ten helices, seventeen strands, and twenty-seven coils. Similarly, H139R and G129R increased strand (seventeen) and coil (twenty-six). In contrast, L70P and R130Q reduce strands (fifteen) and coil (twenty-six), indicating potential structural destabilization. Variants such as H61D, R173H, and D252G exhibit no significant deviation from the wild-type structure (Supplementary Table S2).

We used I-TASSER to predict the 3D structures of wild type and variants to examine the structural variation, which were validated using PROCHECK (Supplementary Fig. S4). Ramachandran plot demonstrated the most of the variants had a high percentage of residues in favored regions, ranging from 92.3% to 93.4%, with minimal residues in disallowed regions (0.5%–1.1%). However, our analysis revealed the mutant G129E exhibited a substantial reduction in structural quality, with only 79.4% of residues in the most favored region (Table 3).

**Table 3:** 3D Model Evaluation of wild type and variant models generated by I-TASSER.

**Substitution**	**I-TASSER** **Score**	**PROCHECK Ramachandran plot analysis**			
	**C-score**	**Residues in most favoured regions**	**Residues in additional allowed regions**	**Residues in generously allowed regions**	**Residues in disallowed regions**
PTEN	-1.19	334(92.0%)	23(6.3%)	2(0.6%)	4(1.1%)
M35R	-1.09	335(92.3%)	22(6.1%)	2(0.6%)	4(1.1%)
H61D	-0.83	335(92.3%)	23(6.3%)	2(0.6%)	3(0.8%)
L70P	-0.90	334(92.3%)	22(6.1%)	3(0.8%)	3(0.8%)
C124R	-1.18	339(93.4%)	18(5.0%)	2(0.6%)	4(1.1%)
G129E	-1.09	289(79.4%)	63(17.3%)	2(0.5%)	10(2.7%)
R130G	-1.11	334(92.3%)	23(6.3%)	2(0.6%)	3(0.8%)
R130Q	-1.07	337(92.8%)	20(5.5%)	2(0.6%)	4(1.1%)
G132V	-0.95	336(92.3%)	21(5.8%)	5(1.4%)	2(0.5%)
R173C	-1.04	337(92.8%)	20(5.5%)	3(0.8%)	3(0.8%)
R173H	-1.19	336(92.3%)	21(5.8%)	3(0.8%)	3(0.8%)
D252G	-0.80	339(93.4%)	17(4.7%)	3(0.8%)	3(0.8%)

Further, structural alignment as assessed by TM-align indicated the majority of variants maintain TM-scores ranging from 0.99499 to 0.99664, indicating alignment with the native protein structure. In comparison, the G129E variant had a lower TM-score of 0.81524, indicating substantial structural alterations. Furthermore, G129E had an RMSD of 2.97, indicating a divergence from the native PTEN protein (Table 4).

**Figure 3:**
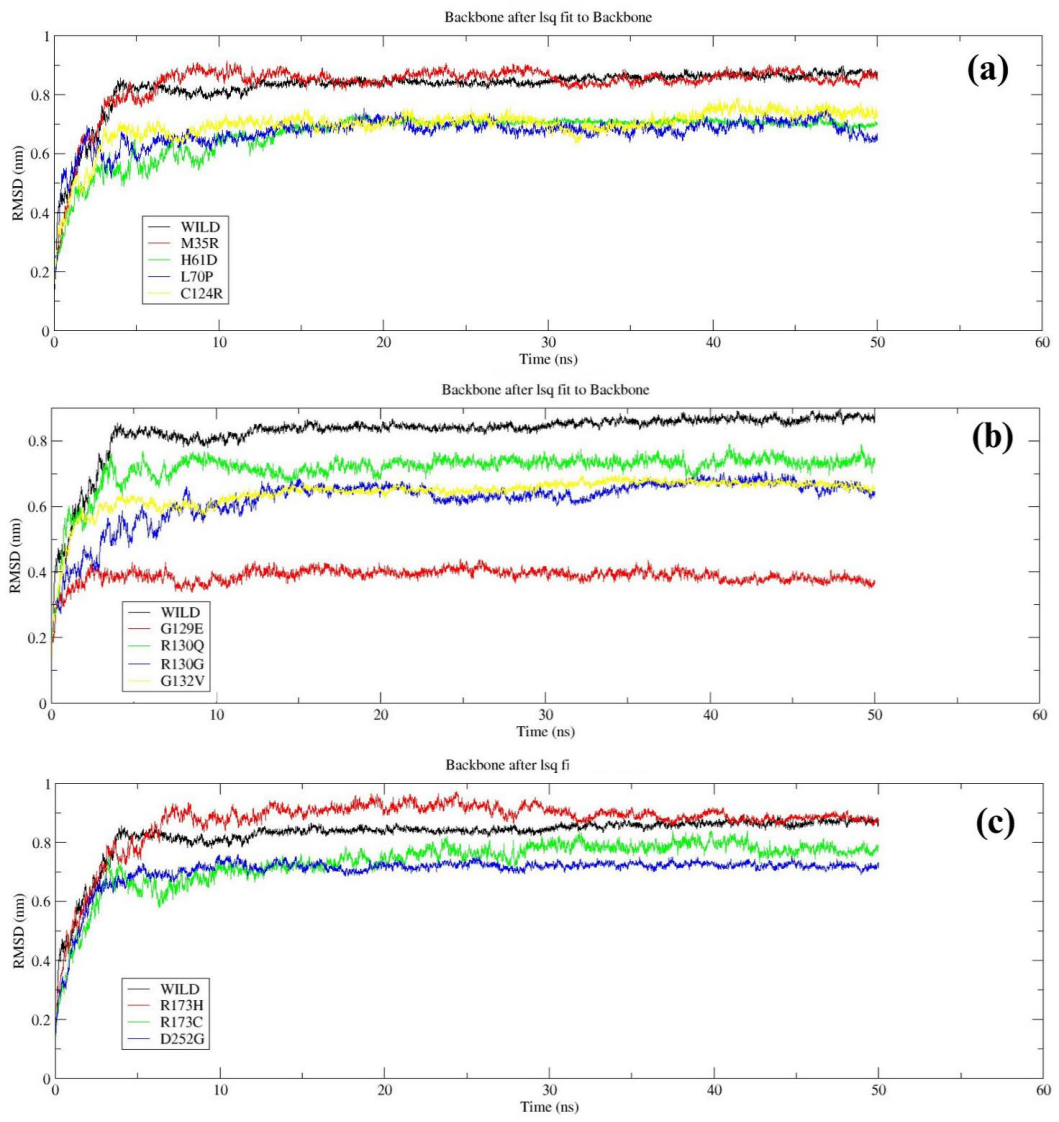
RMSD comparison of Wild-type *PTEN* with mutant variants: (a) Wild-type *PTEN* vs. M35R, H61D, L70P, and C124R; (b) Wild-type *PTEN* vs. G129E, R130Q, R130G, and G132V; (c) Wild-type *PTEN* vs. R173H, R173C, and D252G.

Molecular dynamics simulations was performed using WebGRO to examine the structural stability of the wild-type and mutant proteins over a 50 ns trajectory. RMSD was calculated to assess the structural stability of wild-type and mutant PTEN. The wild-type PTEN had an average RMSD of about ~0.8 nm, indicating a stable structure (Fig. 3a). Mutants such as H61D, M35R, L70P, etc also showed similar stability as the wild type with only slight deviation (~0.6-0.8 nm). Mutants such as G129E displayed lower RMSD (~0.4 nm), suggesting major deviation (Fig. 3b). R173H, R173C and D252G mutants displayed similar stability to the wild type, indicating slight deviations (Fig. 3c).

**Table 4:** TM align analysis of wild type and variant model.

**Substitution**	**RMSD**	**TM-score**
M35R	0.47	0.99590
H61D	0.43	0.99654
L70P	0.46	0.99603
C124R	0.48	0.99579
G129E	2.97	0.81524
R130G	0.52	0.99502
R130Q	0.53	0.99499
G132V	0.46	0.99601
R173C	0.51	0.99518
R173H	0.42	0.99664
D252G	0.48	0.99569

**Figure 4:**
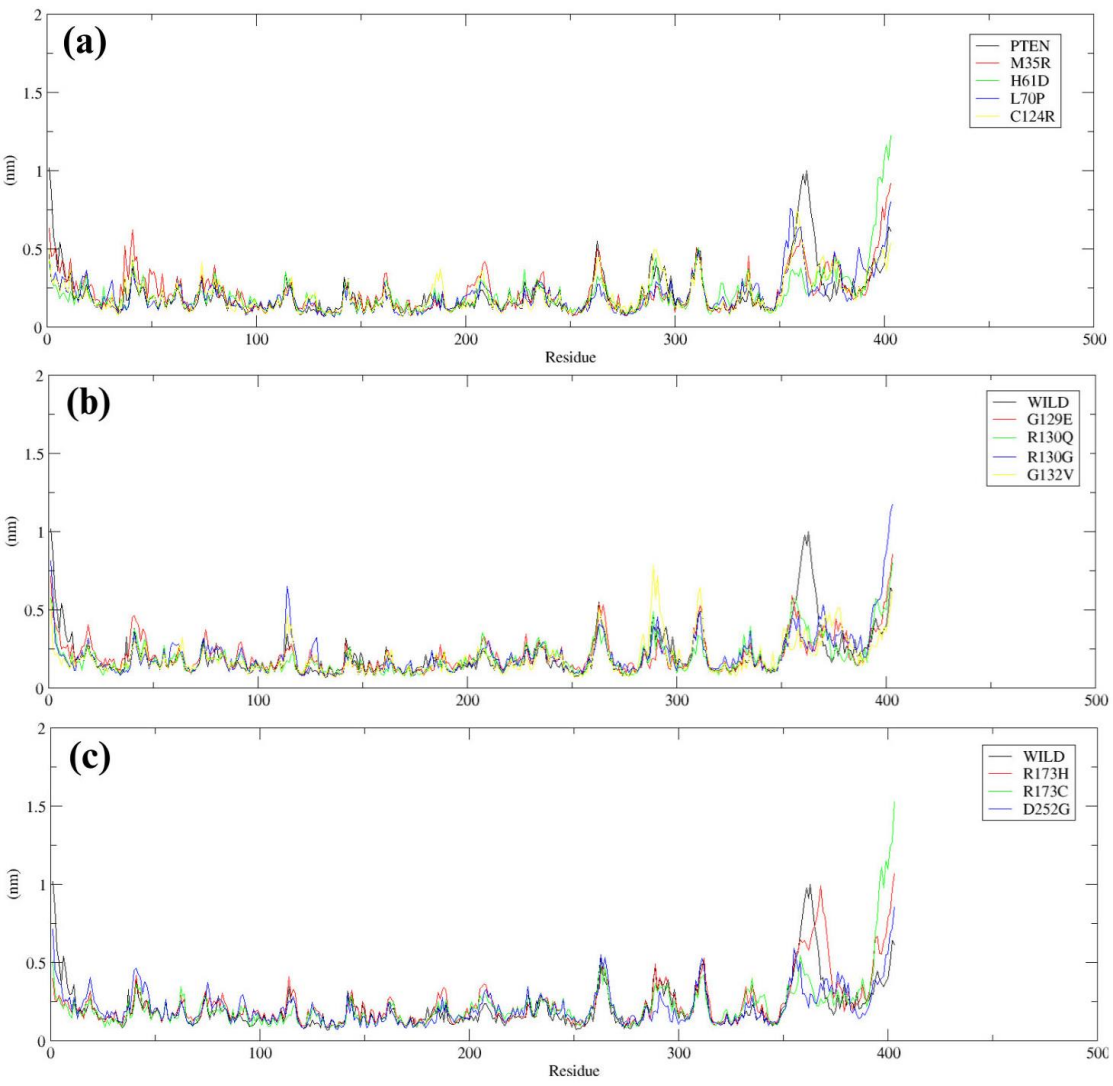
RMSF comparison of Wild-type *PTEN* with mutant variants: (a) Wild-type *PTEN* vs. M35R, H61D, L70P, and C124R; (b) Wild-type *PTEN* vs. G129E, R130Q, R130G, and G132V; (c) Wild-type *PTEN* vs. R173H, R173C, and D252G.

RMSF was calculated to study flexibility in mutants. Wild-type PTEN showed consistent RMSF values, with most residues giving values around 0.5 nm, except for peaks in the loop regions where values reached approximately 1.2 nm (Fig. 4a). For the mutants, M35R and H61D showed a close resemblance to the wild-type profile, with RMSF values remaining below 0.6 nm in most regions. In contrast, mutants like G129E and G132V recorded RMSF peaks much higher than those of the wild type; the highest values of 1.5 nm and above were exhibited particularly in the loop and binding regions (Fig. 4b), indicating increased residue mobility and potential local destabilization. Similarly, R173H, R173C, and D252G mutants exhibited fluctuations in specific regions, with R173H and R173C showing slightly increased RMSF values in flexible loop regions, while D252G displayed the highest fluctuations in key structural areas, suggesting altered local flexibility and potential functional consequences (Fig. 4c).

**Figure 5:**
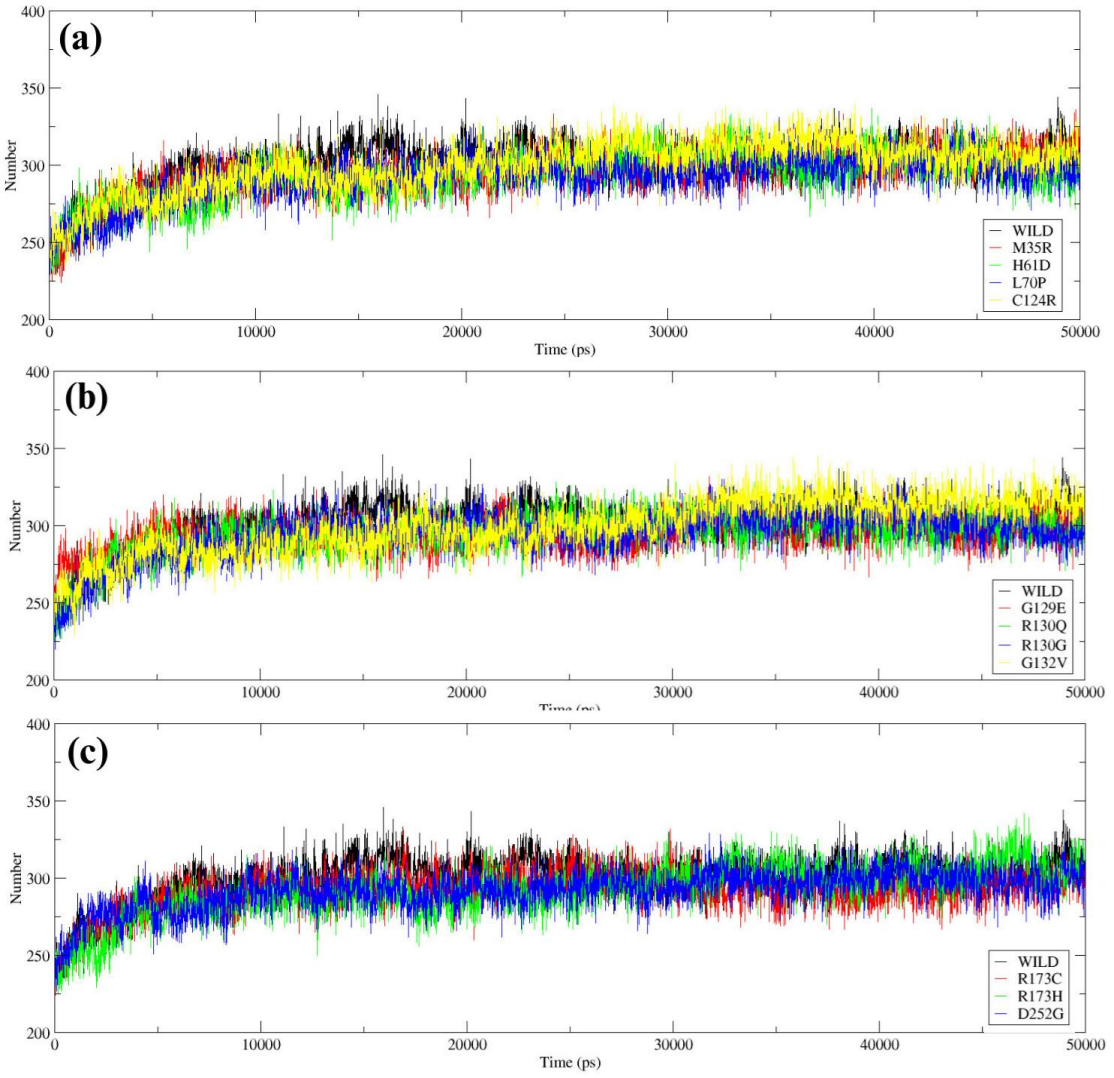
H-bond analysis of Wild-type *PTEN* with mutant variants: (a) Wild-type *PTEN* vs. M35R, H61D, L70P, and C124R; (b) Wild-type *PTEN* vs. G129E, R130Q, R130G, and G132V; (c) Wild-type *PTEN* vs. R173H, R173C, and D252G.

H-bond analysis was performed to measure the stability and structural interactions of wild-type and mutant proteins. The wild-type protein has an average of ~310 H-bonds, indicating structural stability (Fig. 5a). For the mutants, a decline in the number of H-bonds was seen for M35R (H-bonds ~290) meanwhile numbers of H-bonds observed in other mutants were similar with respect to H-bonds in wild PTEN. G129E and G132V mutations exhibited slightly decreased H-bonds (~300–305), signaling slight disruption in structural interactions (Fig. 5b). It can be observed that R173H and R173C mutants gave slight decreases in the order of (~300–305). However, D252G showed the highest decrease (~290), therefore indicating altered interactions (Fig. 5c). 

**Figure 6:**
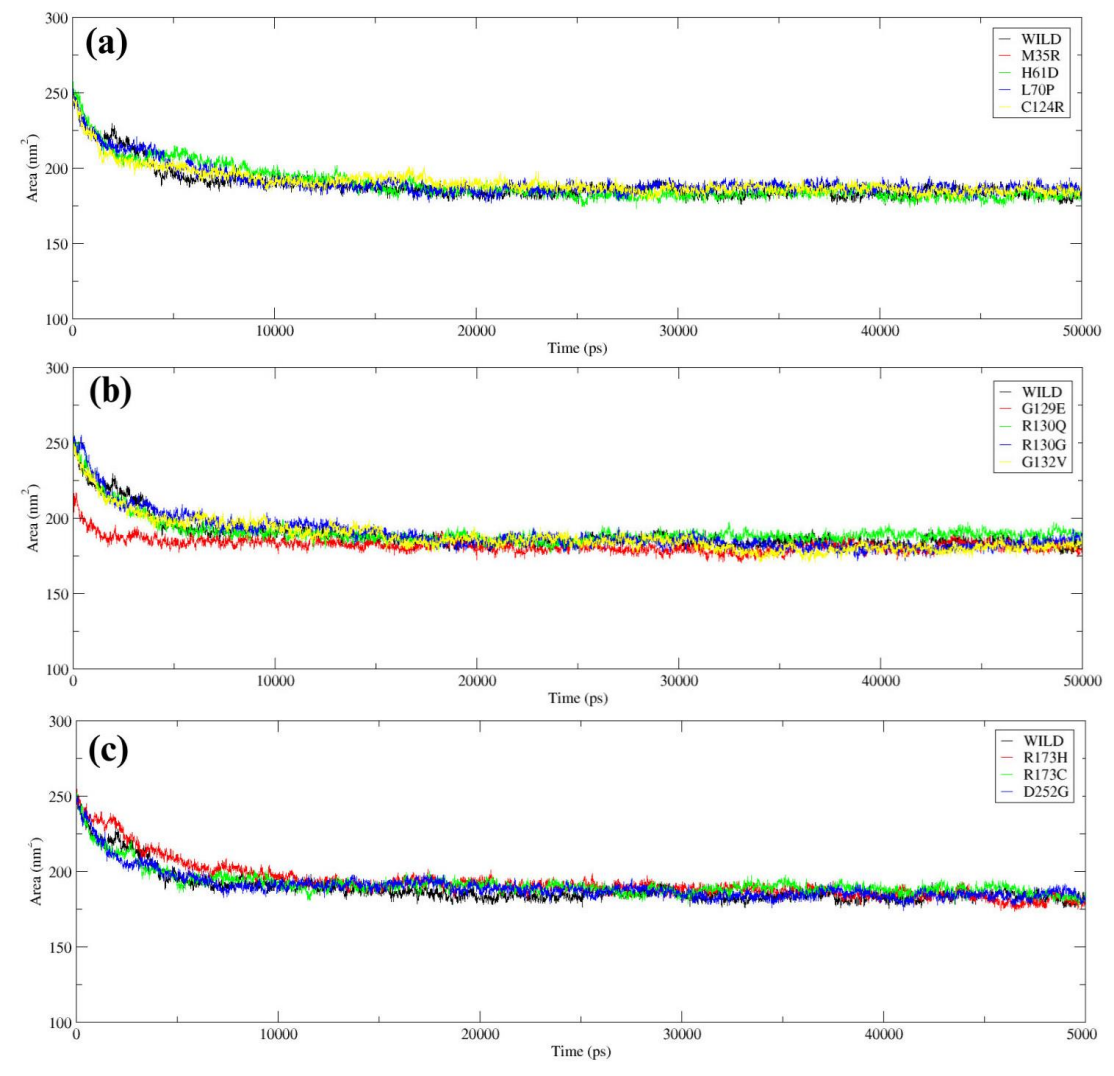
SASA analysis of Wild-type *PTEN* with mutant variants: (a) Wild-type *PTEN* vs. M35R, H61D, L70P, and C124R; (b) Wild-type *PTEN* vs. G129E, R130Q, R130G, and G132V; (c) Wild-type *PTEN* vs. R173H, R173C, and D252G.

SASA analysis was used for the evaluation of the compactness and conformational changes of both the wild-type and mutant PTEN. Wild-type exhibited a fairly consistent decrease in SASA with a steady level of about 200 nm², indicating stable and more compact behaviour. Out of all mutants, C124R had a SASA (~210 nm²), indicating enhanced instability, while all the other mutants possessed SASA values similar to the wild type (Fig. 6a). The mutant G129E showed SASA values lower than the wild type, indicating more compactness. R130G showed a small increase (~205 nm²), indicating slight structural instability (Fig. 6b). R173C showed slightly increased SASA (~205 nm²), indicating loss of conformity (Fig. 6c).

To provide a comparative visualization of hydrogen bonding, solvent accessibility, and compactness, box plot analyses were generated to highlight their variations among wild-type PTEN and its mutants. The comparative box plot analysis for hydrogen bonds revealed that mutants such as D252G, G129E, R173C, and M35R exhibited a lower number of hydrogen bonds compared to wild-type PTEN, indicating potential structural destabilization. SASA analysis showed that G129E had reduced solvent accessibility compared to the wild type, whereas H61D and R173H displayed increased SASA values, suggesting altered structural exposure. The box plot analysis for Rg further indicated that G129E and G132V had lower Rg values, suggesting a more compact structure, while R130Q, R173C, and R173H exhibited a slight increase in Rg, indicating a potential loss of conformational stability (Supplementary Fig. S5a-c).

**Figure 7:**
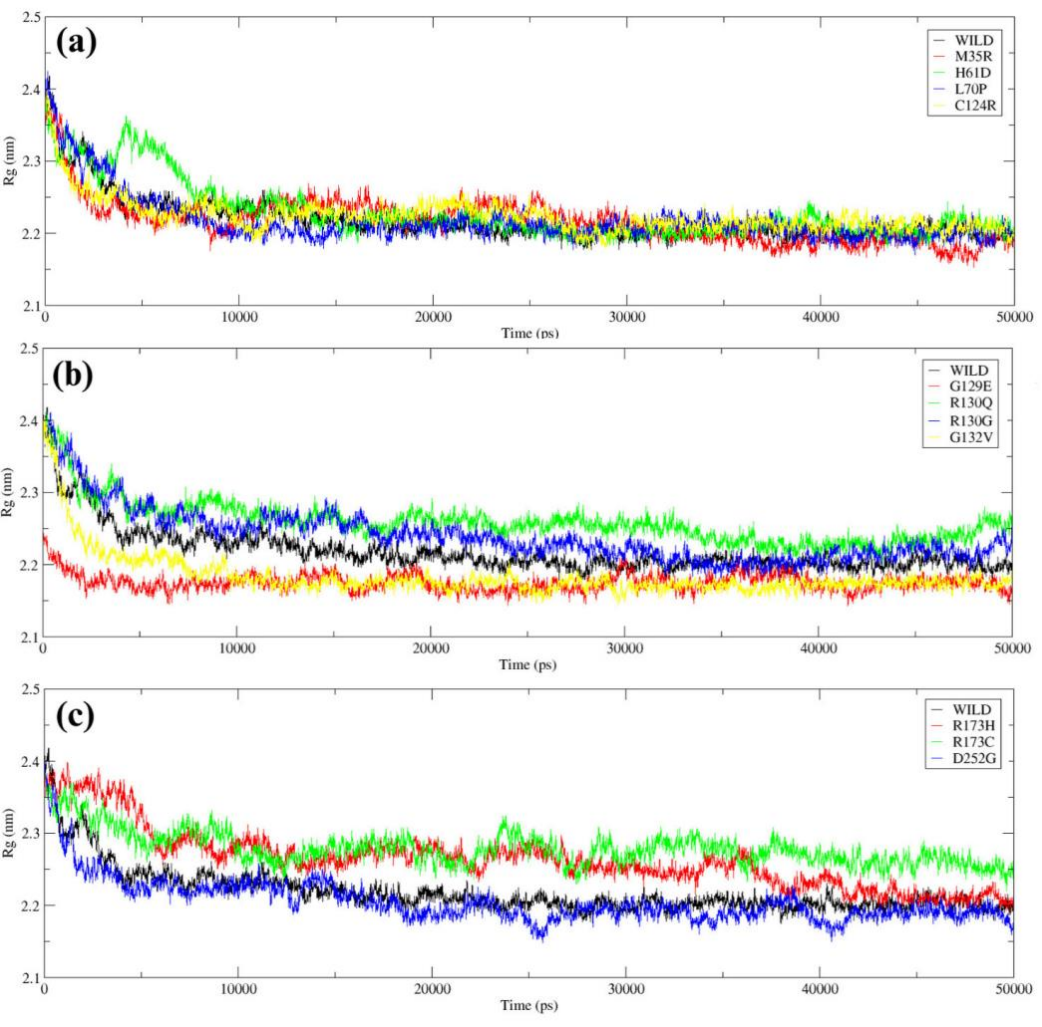
The Rg of (a) Wild *PTEN* vs M35R, H61D, L70P and C124R, (b) Wild *PTEN* vs G129E, R130Q, R130G and G132V and (c) Wild *PTEN* vs R173H, R173C and D252G.

The Rg analysis indicates the compactness and stability of the wild-type and mutant PTEN structures. The wild-type exhibited a consistent and stable Rg, with an average of ~2.3 nm, confirming its compact and stable conformation. When compared to the wild type, other mutants, among them M35R, H61D, and L70P, all exhibited similar Rg, signifying that there are no substantial variations in compactness in comparison to wild type (Fig. 7a). G129E and G132V show a lower Rg of ~2.18 nm, which implies the structure is more compact than the wild type (Fig. 7b). R173C and R173H exhibits a little higher Rg of ~2.23 nm, signifying loss of conformational stability (Fig. 7c).

## DISCUSSION

SNPs are essential to understanding the genetic basis of complex disorders. However, identifying functional SNPs in disease-associated genes remains a significant challenge [43]. Among them, nsSNPs are particularly important as they result in amino acid substitutions that can alter protein structure and function, potentially leading to disease development [44]. Several studies have linked missense mutations, insertions, and deletions to various cancers, underscoring their role in oncogenesis [45]. 

Our study focuses on the impact of impact of deleterious nsSNPs on the structure and function of PTEN, a key tumor suppressor gene frequently mutated in human tumors [46]. In bioinformatics approaches various tools and algorithms is employed to analyze proteins and gene related disorders. However, to enhance prediction accuracy, it is recommended to use multiple predictive tools and establish a consensus by cross-validating their outputs [47-50]. Similar bioinformatics-driven approaches have been widely used in cancer research to investigate other molecular alterations, such as miRNA dysregulation and its impact on key oncogenic pathways [51, 52]. In our study, we focused on nsSNP-induced structural changes, analyzing 1,434 nsSNPs out of a total of 43,855 SNPs in the *PTEN* gene using six computational tools to identify deleterious variants. A total of seventeen nsSNP were screened and were selected for structural and functional study after being found to be deleterious in five out of six tools.

To evaluate the effect of mutations on protein stability, we employed MUpro, I-Mutant 2.0, and INPS-MD to calculate free energy changes. The results indicated that fifteen out of seventeen mutations decreased protein stability, potentially leading to functional impairment. Given that protein stability is critical for maintaining proper folding, structural integrity, and biological function, these findings suggest that destabilizing mutations in PTEN may contribute to its tumor-suppressive dysfunction.

The HOPE server further demonstrated the impact of mutations on the structural and functional properties of the proteins. The protein core becomes unstable due to mutations like M35R and G129E, which introduce buried charges, whereas replacements like H61D, I135T, and R173C involve smaller residues and result in the loss of important connections. Proline alterations (L70P, L112P) were predicted to cause structural destabilization by disrupting alpha helices, while bigger mutant residues (H93R, C124R) were implicated in improper folding and molecular interaction. Mutations such as D107N, R130Q, and R173H resulted in loss of charge, compromising interaction networks. Previous studies have shown that such alterations in electrostatic properties can severely impact protein function and interactions [53]. 

Conservation analysis of mutants was further studied by Consurf which assigned the highest score of nine to the twelve variants and categorized them into buried structural and exposed functional residues. The ConSurf conservation score is a relative measure based on evolutionary constraints rather than an absolute similarity percentage [33, 54]. Mutants M35R, L70P, C124R and G132V are buried structural residues that disrupt protein stability by altering hydrophobic core, whereas H61D, H93R, G129E, R130G, R130Q, R173C, R173H, and D252G are exposed functional residues which impact the molecular interaction. The mutation in buried resides can affect the structural integrity of the protein whereas the polymorphism in exposed resides may alter the protein function [55].

We further explored PTEN’s functional interactions using the STRING database, which highlighted PTEN’s central role in the PI3K/AKT signaling pathway and its cooperation with TP53 in tumor suppression. Disruptive mutations in PTEN may impair its ability to bind key regulatory proteins such as PIK3CA, AKT1, and TP53, leading to dysregulation of PI3K/Akt signaling. Additionally, CDD analysis showed that mutations C124R, R130G, and R130Q led to the loss of active and catalytic sites, further suggesting compromised enzymatic function [56].

In addition, the observed changes in secondary structure suggest that nsSNPs can impact PTEN’s function by altering its stability and enzymatic activity. The increase in beta-strands and coils in C124R and R130G may lead to structural flexibility that disrupts PTEN’s catalytic conformation, while the reduction in strands and coils in L70P and R130Q may cause destabilization, affecting protein interactions and localization [57]. Mutants with minimal secondary structure deviations are less likely to significantly impact PTEN’s function though their effects may still require further experimental validation.

The three-dimensional structural information of the protein enhances the accuracy of identifying deleterious amino acid substitutions and provides valuable insights into associated molecular alterations [58]. In this study, the 3D structural analysis of PTEN and its mutants was performed using I-TASSER, followed by refinement with GalaxyRefine [37, 59]. Given the incomplete nature of available experimentally determined PTEN structures, I-TASSER was selected to generate a more complete model. Structural validation using PROCHECK confirmed that the majority of variants retained a high percentage of residues in the most favored regions of the Ramachandran plot (>92%), with minimal residues in disallowed regions (<1.1%). Among the analyzed mutations, G129E exhibited the most pronounced deviation, with only 79.4% of residues in the favored region, suggesting significant conformational alterations compared to other mutations [40]. To further evaluate structural deviations, we performed 3D structural alignment using TM-align. Most mutant structures showed high similarity to the wild-type, with TM-scores exceeding 0.99 and RMSD values below 0.53. However, G129E exhibited a significantly lower TM-score of 0.81524 and a higher RMSD of 2.97, suggesting substantial conformational changes that could impair PTEN’s function. These structural changes may lead to misfolding and disrupt PTEN’s phosphatase activity, ultimately affecting its ability to regulate key cellular signaling pathways [60].

Molecular dynamics (MD) simulations provide deeper insights into the behavior of proteins under physiological conditions [61]. This approach offers the best correlation with experimental studies, allowing for a more accurate assessment of protein stability and dynamics [62]. Since protein attributes such as flexibility, compactness, and hydrogen bonding are interconnected, analyzing them collectively is essential when investigating structural effects of mutations [63].

To evaluate protein stability and flexibility, we analyzed RMSD and RMSF during simulations. RMSD analysis revealed that mutants R173C, G129E, and D252G exhibited higher deviations compared to the wild type, indicating potential destabilization. RMSF analysis showed that M35R and H61D had similar fluctuations to the wild type, suggesting minimal impact on flexibility, whereas G129E, R130G, and R173C exhibited higher peaks, indicating increased mobility and structural instability at the residue level [64]. 

The hydrogen bond analysis indicates that the wild-type protein maintained a constant H-bonding network during the simulation and hence asserted its structural stability. The differences in hydrogen bonds among the mutants indicate some degree of destabilization due to mutations, being most significant for M35R and D252G [65]. The SASA analysis was conducted to evaluate the compactness and conformational changes in the wild-type and mutant PTEN proteins. Overall, the wild type maintained a compact and stable conformation, while the mutants showed similar results to that of wild type, and mutations G129E and G132V were that showed lower Rg values also indicating deviation. Other Rg values were very close to those of the wild type and showed a low difference in stabilities [66]. 

In conclusion, our study provides a comprehensive in silico analysis of deleterious nsSNPs in the *PTEN* gene, elucidating their structural and functional impacts. Among 17 identified variants, mutations such as G129E, C124R, and R173H significantly disrupted PTEN stability and enzymatic activity, affecting its ability to regulate the PI3K/Akt pathway. Structural alignment and molecular dynamics simulations highlighted G129E as a major destabilizing mutation with significant conformational deviations. This underscores the critical role of both buried and exposed residues in maintaining PTEN's structural integrity and biological function. The findings emphasize the utility of integrating multiple computational tools for accurate prediction and analysis, paving the way for targeted experimental studies and potential therapeutic strategies to mitigate the effects of deleterious mutations in PTEN.
