# Supplementary material for: Comprehensive computational analysis of deleterious nsSNPs in PTEN gene for structural and functional insights
Source: Mol Biol Res Commun. 2025;14(3):219–39. doi: 10.22099/mbrc.2025.52148.2092 (PMC12046362; doi:10.22099/mbrc.2025.52148.2092)
Supplement: Supplementary file 1 — Tables S1-S2 [file MBRC-14-219-s001.pdf]

**Supplementary Table S1:** Property Analysis by Project HOPE.

| Variants | Wild type | Mutant type | Biological Effect |
|---|---|---|---|
| M35R | 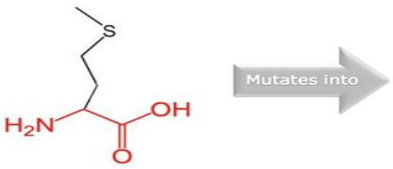 Neutral | 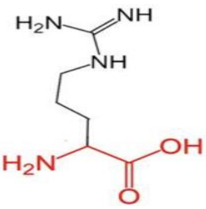 Positive | The mutant residue introduces a charge in a buried region, potentially leading to protein folding issues and loss of interactions. The wild-type residue is more hydrophobic. |
| H61D | 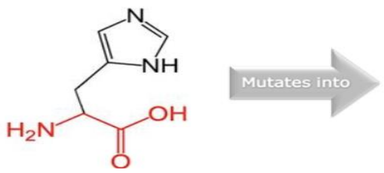 Neutral | 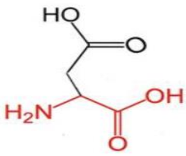 Negative | The mutant residue is smaller, which may result in the loss of interactions with other molecules, possibly affecting external interactions. |
| L70P | 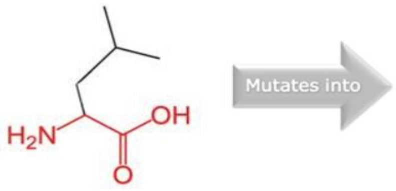 | 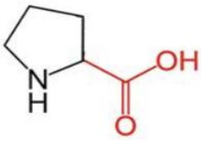 | The mutation creates a void in the protein core, potentially destabilizing the structure. |
| H93R | 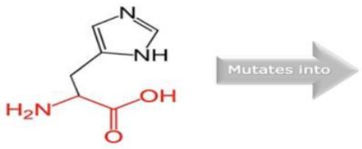 Neutral | 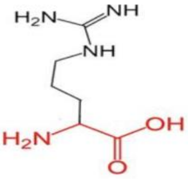 Positive | The mutant residue is larger, disrupting interactions with other molecules or different regions of the protein. |
| D107N | 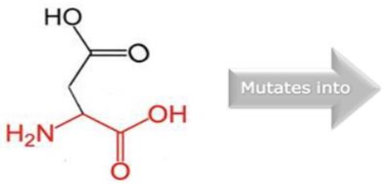 gative | 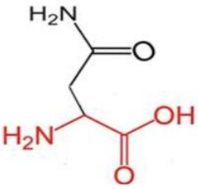 Ne Neutral | The mutation leads to the loss of charge in the wild-type residue, which may disrupt interactions with other molecules. |

| | | | | |
|---|---|---|---|---|
| L112P | 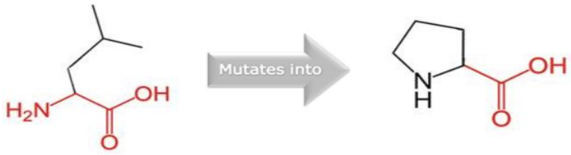 | | | Proline disrupts an α-helix; this mutation may destabilize the helix, severely affecting protein structure. |
| C124R | 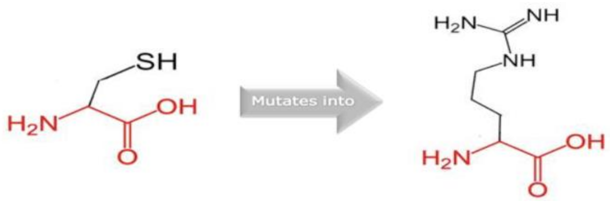 | Neutral | Positive | The mutation disrupts hydrophobic interactions in the protein core, potentially leading to folding issues. The wild-type residue is more hydrophobic. |
| G129E | 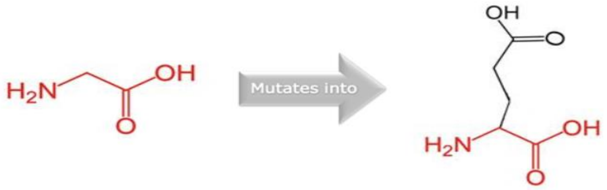 | Neutral | Negative | The mutant residue is larger and introduces a charge in a buried region, which may cause protein folding problems. The wild-type residue is more hydrophobic. |
| R130G | 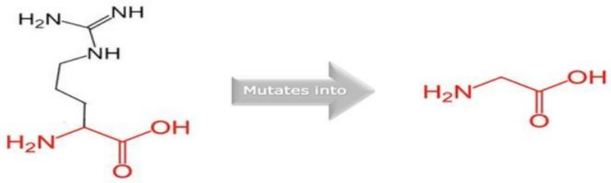 | Positive | Neutral | Glycine's flexibility can compromise the necessary rigidity of the protein at this position. The wild-type residue is more hydrophobic. |
| R130Q | 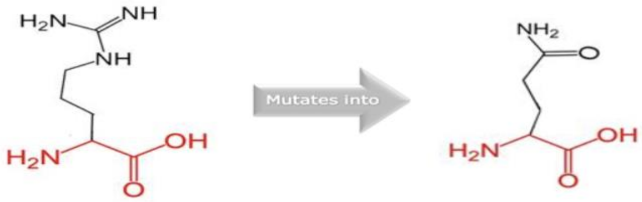 | Positive | Neutral | The mutation eliminates the charge of the buried wild-type residue, creating a void in the protein core. |
| G132V | 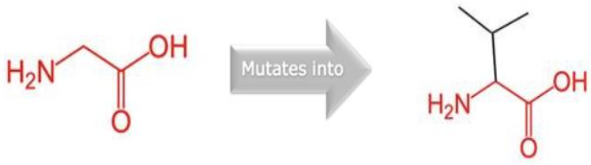 | | | The glycine mutation forces the local backbone into an incorrect conformation, disrupting the local structure. The wild-type residue is more |

| | | | | |
|---|---|---|---|---|
| | | | | hydrophobic. |
| I135T | 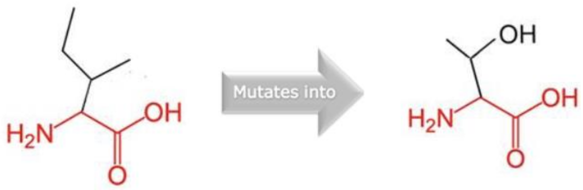 | | | The mutant residue is smaller, potentially leading to a loss of hydrophobic interactions in the protein core. The wild-type residue is more hydrophobic. |
| R173C | 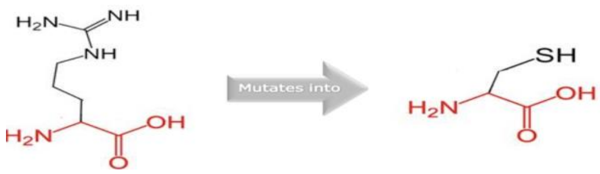 | Positive | Neutral | The mutant residue is smaller, which may result in the loss of interactions with other molecules, possibly affecting external interactions. The wild-type residue is more hydrophobic. |
| R173H | 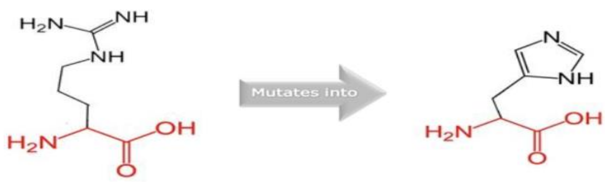 | Positive | Neutral | The mutation results in the loss of the wild-type residue's charge, affecting interactions with other molecules or residues. |
| D252G | 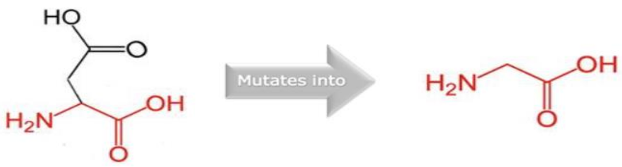 | Negative | Neutral | Glycine's flexibility can disrupt the required rigidity of the protein at this position. The wild-type residue is more hydrophobic. |

**Supplementary Table S2:** Predicted secondary structure of each amino acid residue in mutants and native.

| Wild Type and Variants | No. of Helices | No. of Strands | No. of Coil |
|---|---|---|---|
| PTEN (WT) | 9 | 16 | 25 |
| M35R | 10 | 15 | 25 |
| H61D | 9 | 16 | 25 |
| L70P | 9 | 15 | 24 |
| H93R | 9 | 17 | 26 |
| C124R | 9 | 18 | 27 |
| G129E | 9 | 17 | 26 |
| R130G | 10 | 17 | 27 |
| R130Q | 10 | 15 | 24 |
| G132V | 10 | 16 | 26 |
| R173C | 9 | 17 | 26 |
| R173H | 9 | 16 | 25 |
| D252G | 9 | 16 | 25 |
