# Supplementary material for: Comprehensive computational analysis of deleterious nsSNPs in PTEN gene for structural and functional insights
Source: Mol Biol Res Commun. 2025;14(3):219–39. doi: 10.22099/mbrc.2025.52148.2092 (PMC12046362; doi:10.22099/mbrc.2025.52148.2092)
Supplement: Supplementary file 2 — Figures S1-S5 [file MBRC-14-219-s002.pdf]

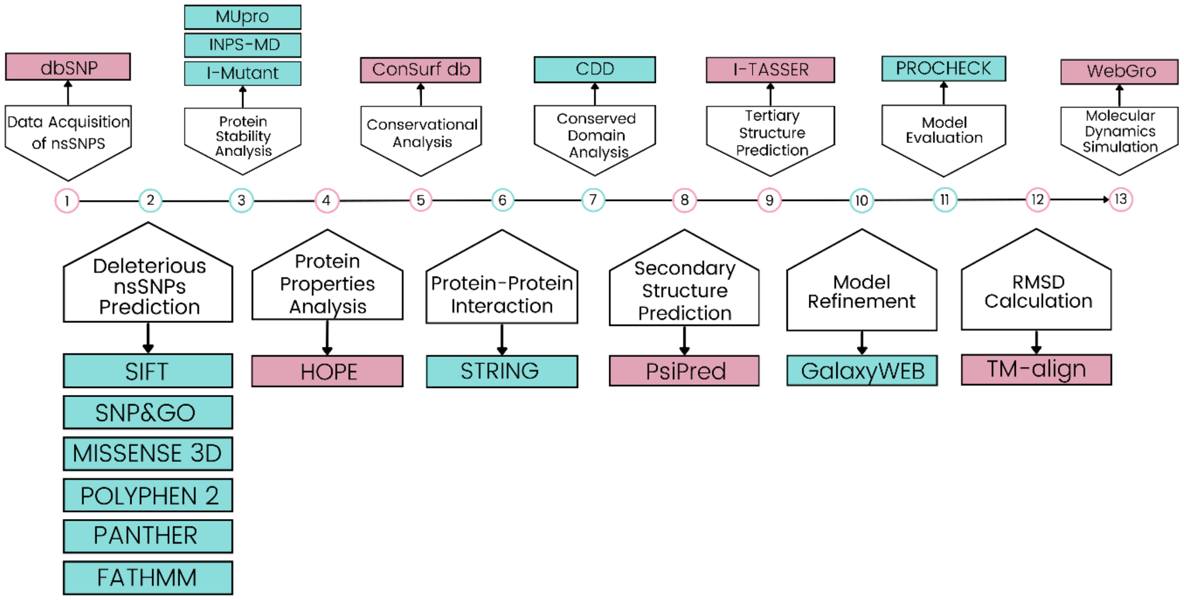

Figure S1: Workflow of methodology.

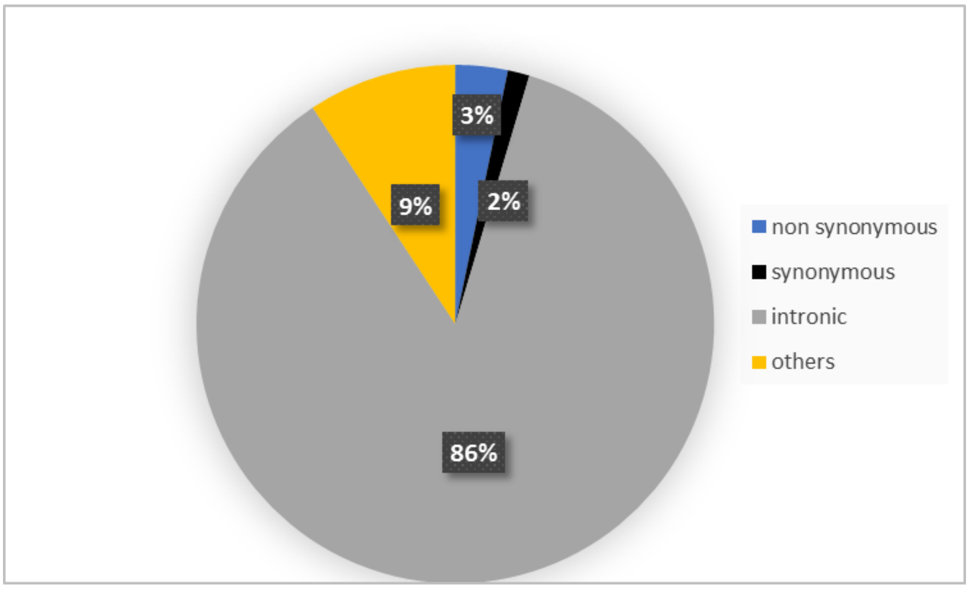

Figure S2: SNP distribution within the PTEN gene.

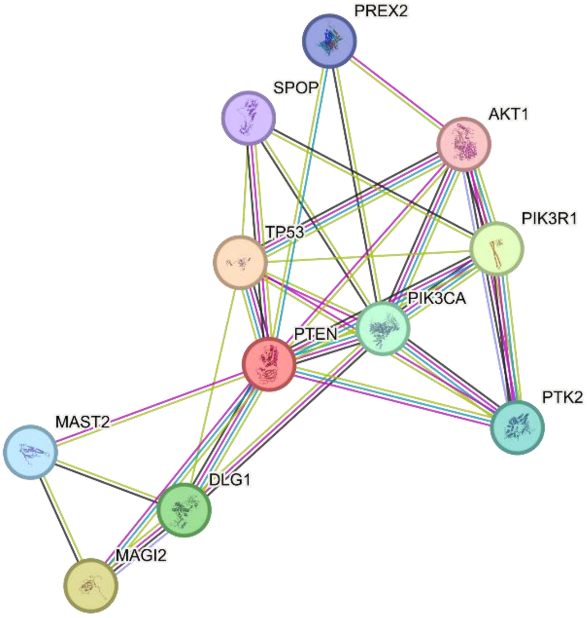

Figure S3: Predicted functional partners of PTEN.

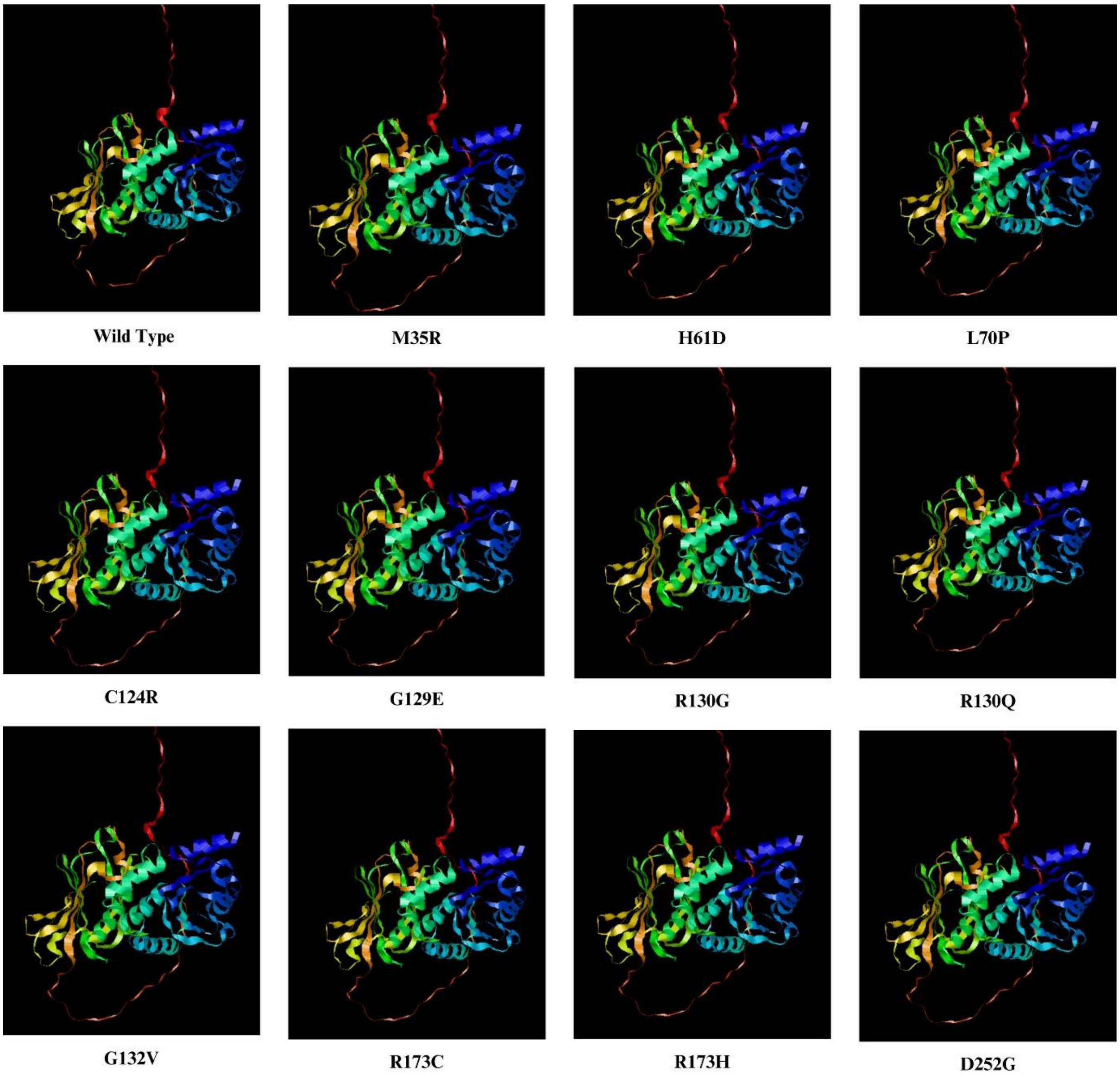

Figure S4: 3D Structure of wild type PTEN and its Mutants

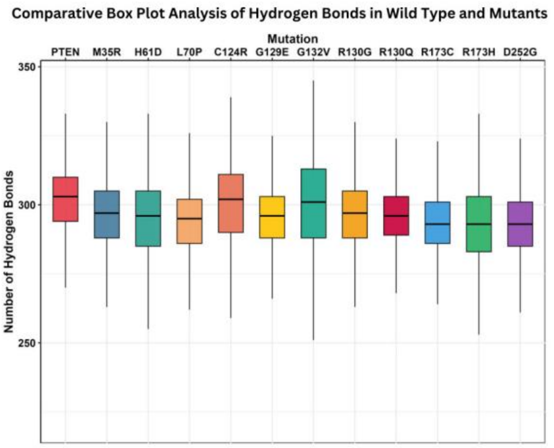

(a)

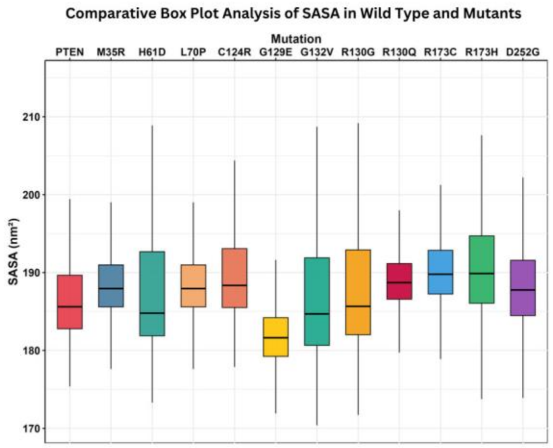

(b)

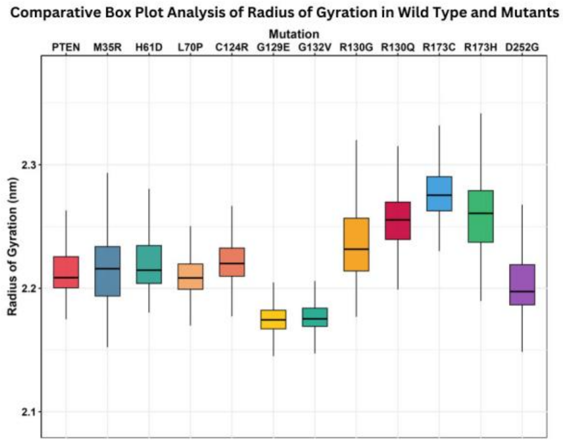

(c)

Figure S5: Box plot analysis of hydrogen bonds, SASA, and Rg in wild-type PTEN and its mutants, highlighting structural variation(a) Reduced hydrogen bonds in G129E, R130Q, and R173H suggest destabilization. (b) Increased SASA in G129E and R130Q indicates altered solvent exposure, while L70P and R173H show reduced SASA, suggesting compaction. (c) Higher Rg in G129E and R173H implies expansion, whereas L70P and G132V exhibit greater compactness.
